# *Rhaponticum carthamoides* transformed root extract inhibits human glioma cells viability, induces double strand DNA damage, H2A.X phosphorylation, and PARP1 cleavage

**DOI:** 10.1007/s10616-018-0251-3

**Published:** 2018-08-31

**Authors:** Ewa Skała, Monika Toma, Tomasz Kowalczyk, Tomasz Śliwiński, Przemysław Sitarek

**Affiliations:** 1 Department of Biology and Pharmaceutical Botany, Medical University of Łódź, Muszyńskiego 1, 90-151 Łódź, Poland; 2 Laboratory of Medical Genetics, Faculty of Biology and Environmental Protection, University of Łódź, Pomorska 141/143, 90-236 Łódź, Poland; 3 Department of Genetics, Plant Molecular Biology and Biotechnology, Faculty of Biology and Environmental Protection, University of Łódź, Banacha 12/16, 90-237 Łódź, Poland

**Keywords:** *Rhaponticum carthamoides*, Caffeoylquinic acid derivatives, Transformed roots, Glioma cells, PARP1 cleavage, H2A.X phosphorylation

## Abstract

*Rhaponticum carthamoides* transformed root extract induces double strand DNA damage by increasing the number of phosphorylated H2A.X- and cleaved PARP1-positive U87MG cells and patient-derived IV grade glioma cells. Furthermore, treatment of these cells with root extract causes down-regulation of *UHRF1* and *DNMT1*. Transformed root extract is rich in caffeoylquinic acid derivatives, especially tricaffeoylquinic acid derivatives. Our findings demonstrate that the *R. carthamoides* transformed root extract may trigger apoptosis in glioma cells by induction of DNA damage, PARP cleavage and epigenetic modification.

**Electronic supplementary material:**

The online version of this article (10.1007/s10616-018-0251-3) contains supplementary material, which is available to authorized users.

## Introduction

Glioblastoma is a type of glioma and is classified by the World Health Organization (WHO) as grade IV astrocytoma. Glioblastoma is one of the most common, aggressive and lethal forms of cancer (Li et al. 2015; Furnari et al. 2015), partly due to the impossibility of total surgical resection (Eom et al. 2010), as well as resistance to chemotherapy and the impermeability of the blood–brain barrier (Li et al. 2015). Moreover, synthetic drugs are highly toxic (Khan et al. 2015). For many years, research has attempted to identify novel strategies that avoid the use of toxic chemotherapy which causes the death of healthy cells alongside the target cancer cells. Natural compounds derived from microorganisms, marine organisms and plants are very promising sources for cancer treatment (Khan et al. 2015). Some of them exhibit low toxicity, reduced side effects and are inexpensive (Kuno et al. 2012; Seca and Pinto 2018). More than 3000 plant species are known to possess anticancer activity against various types of cancer cells (Tariq et al. 2017; Seca and Pinto 2018). One such species is *Rhaponticum carthamoides*, a member of the Asteraceae family. The raw materials of this species are roots and rhizomes which contain ecdysteroids, triterpenoids, polyacetylenes, flavonoids and phenolic acids with caffeoylquinic acid derivatives (Kokoska and Janovska 2009; Skała et al. 2015). Previous studies have demonstrated that the roots of this species inhibit the viability of MCF-7 human breast cancer cells (Hamburger et al. 2006), and *R. carthamoides* transformed roots obtained by *Agrobacterium rhizogenes* transformation suppressed the viability of grade II, III and IV patient-derived glioma cells by inducing apoptosis via the intrinsic pathway and caspase activation (Skała et al. 2016, 2018).

The present study examines the mechanism of the anticancer effect of *Rhaponticum carthamoides* transformed roots extract (*Rc* TR extract) against grade IV patient-derived human glioma cells and U87MG cells. Its aim was to confirm whether the *Rc* TR extract could inhibit the glioma cells viability and induce apoptosis by increasing the number of cleaved Poly(ADP-ribose) (PARP1)-positive cells, inducing DNA damage, thus altering the level of phosphorylated H2A.X-positive cells a marker of double strand breaks in DNA. PARP1, polymerase is a nuclear NAD^+^-dependent enzyme activated in response to DNA damage which plays role in transfer ADP-ribose to itself and other nuclear proteins (Gobeil et al. 2001; Kim et al. 2005). PARP1 is responsible for DNA repair, DNA stability and transcriptional regulation (Los et al. 2002). In response to caspase activation, the 116 kDa PARP1 protein is cleaved into 85 kDa fragment, which indicates cell apoptosis (Bhouri et al. 2012; Finco et al. 2016; Esposito et al. 2017). In addition, to determine whether *Rc* TR extract could induce the apoptosis of glioma cells, we evaluated the levels of DNA epigenetic markers such as UHRF1 and DNMT1.

## Materials and methods

### Plant material, sample extraction and chemical analysis

*Rhaponticum carthamoides* transformed roots obtained via its transformation by A4 *Agrobacterium rhizogenes* was used as the material. Our earlier study describe the establishment and growth of the transformed roots (Skała et al. 2015). The extraction procedure of lyophilized plant material (10 g dry weight) with 80% (v/v) aqueous methanol was performed as described previously (Skała et al. 2016). The yield (w/w) of the aqueous methanol extract (*Rc* TR extract) was 17.73% (Skała et al. 2016). HPLC–PDA and UPLC-PDA-ESI-MS^3^ methods were used for chemical analysis of the *Rc* TR extract (Skała et al. 2015).

### Cell cultures of glioma cells

In this study was used two cell lines of astrocytoma grade IV: one being the U87MG cell line (89081402, Sigma, St. Louis, MO, USA) and the other obtained from surgical specimens, from a patient. The establishment of the patient-derived glioma cells was described in our earlier study (Skała et al. 2016). The cells were grown in either DMEM (Gibco, Thermo Fisher Scientific, Waltham, MA, USA) or EMEM (EBSS) (Sigma) medium supplemented with 10% Fetal Bovine Serum (EuroClone, Pero MI, Italy), penicillin and streptomycin (Lonza, Basel, Swizerland). The cells were placed at a density of 2–4 × 10^4^ cells/cm^2^ and cultured in accordance with the manufacturer’s protocol (Sigma) at 37 °C in a humidified atmosphere containing 5% CO_2_.

### MTT

The cell viability was measured by MTT assay according to Skała et al. (2016). The human glioma cells were placed into 96-well microplates at 4 × 10^4^ cells/well and incubated with 0.1–3.0 mg/mL of *Rc* TR extract for 24 h.

### Double strand breaks (DSBs): neutral comet assay

To detect double strand breaks (DSBs), the neutral version of the comet assay according to Nieborowska-Skorska et al. (2006) was used with modifications. The glioma cells were treated with 0.25–1.5 mg/mL *Rc* TR extract for 24 h. Then, the cells were washed with PBS, detached from bottle’s surface using TrypLE Express ENzyme (Gibco) and centrifuged. Then, the cells were mixed with 0.75% LMP agarose (Sigma) and spread on microscope slides precoated with 0.5% NMP agarose (Sigma). The cells were then lysed for 1 h at 4 °C in a buffer consisting of 2.5 mM NaOH, 100 mM EDTA, 1% Triton X-100, 10 mM Tris, pH 10. The further procedure was carried out in line with the previous studies (Czyż et al. 2016). The data were measured for each sample from randomly selected 50 cells per slide and are expressed as the mean value ± SD from three independent experiments. DNA damage was quantified by the percentage of DNA in the tail.

### Measurement of phosphorylated H2A.X and cleaved PARP levels

The glioma cells were seeded in a 6-well plate at a density of 2 × 10^5^ cells/well and treated with *Rc* TR extracts (0.75 mg/mL) for 24 h. The cells cultured in the absence of the *Rc* TR extract were used as the control. The phosphorylated H2A.X- and cleaved PARP-positive cells were measured using Apoptosis, DNA Damage and Cell Proliferation Kit (BD Pharmingen, San Jose, CA, USA, 562253) according to the manufacturer’s protocol. In this experiment we used only two compounds of the mentioned kit—Alexa Fluor^®^ 647 Mouse Anti-H2A.X (pS139) and PE Mouse Anti-Cleaved PARP (Asp214) Antibodies. All experiments were performed using a FACS Canto II cytometer (Becton–Dickinson, San Jose, CA, USA).

The level of γ-H2A.X was also measured using an H2A.X Phosphorylation Assay Kit (Millipore, Billerica, MA, USA) according to the protocol of the manufacturer. Chemiluminescence detection was performed using attached HRP-substrates using a GloMax-Multi device (Promega, Madison, WI, USA).

### Real-time PCR analysis

The cells were incubated for 24 h with 0.75 mg/mL of *Rc* TR extract. The control cells were grown in the absence of the plant extract. RNA isolation kit (A&A Biotechnology, Gdynia, Poland) was used to isolate RNA according to the manufacturer’s protocol. cDNA was synthesized from the total RNA using the TranScriba Kit (A&A Biotechnology) according to the manufacturer’s protocol. Real-time PCR was performed using TaqMan^®^ Real-time PCR Master Mix (Life Technologies, Carlsbad, CA, USA) and Agilent Technologies Stratagene Mx300SP with MxPro software. TaqMan probes (Life Technologies) were used to analyze two genes (*UHRF1*, *DNMT1*). As the reference gene was used *18S RNA* (Life Technologies). The qRT-PCR programme was 95 °C for 10 min, 30 cycles of 95 °C for 15 s and 60 °C for 60 s. Each sample was repeated three times. The comparative Ct method was used to calculate relative fold-changes in gene expression and normalized to the average of *18S RNA*.

### Statistical analysis

The results are expressed as means from three independent experiments ± SD. The Shapiro–Wilk test was used to determine the normality of the data. The Kruskal–Wallis test and the one-way analysis of variance (ANOVA) with Tukey’s post hoc test were used to determine the significant differences (*p* < 0.05) between the samples. The statistical analysis was performed using STATISTICA 12.0 software (StatSoft, Krakow, Poland).

## Results

### The glioma cell viability was reduced in a dose-dependent manner after treatment with *Rc* TR extract

**Fig. 1:**
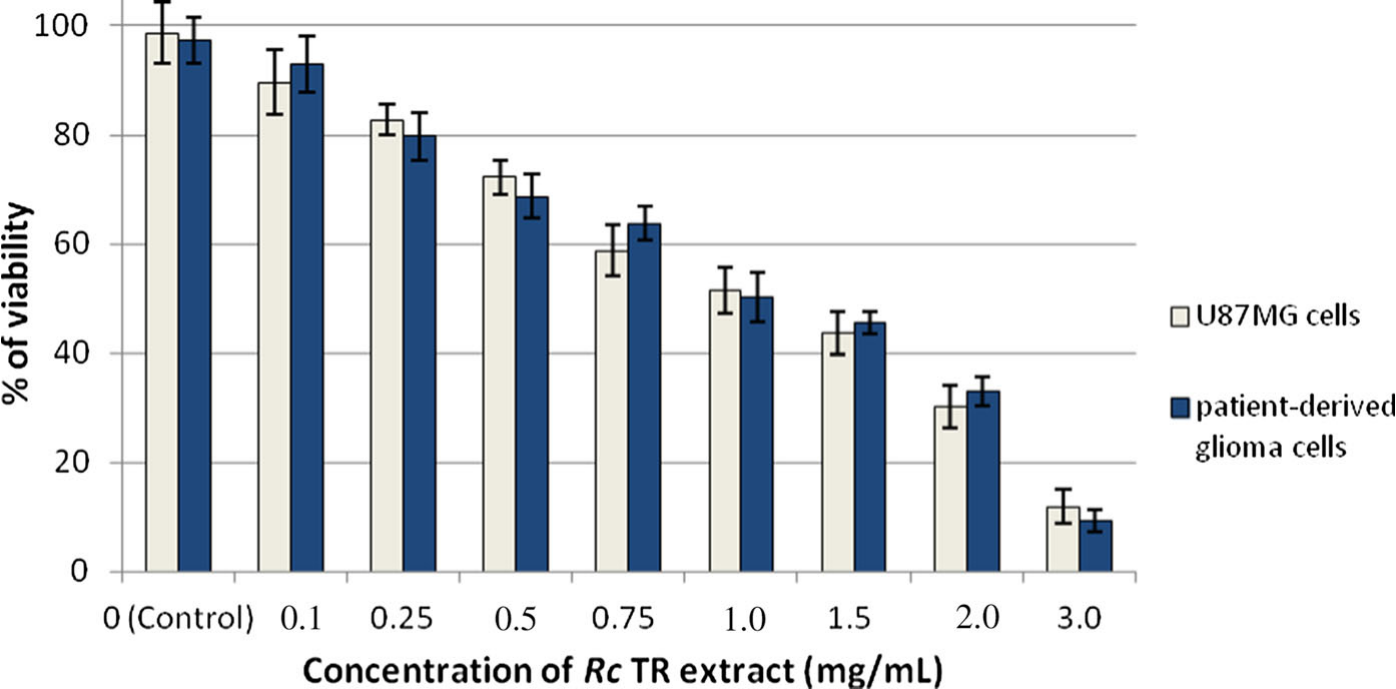
Effect of *Rc* TR extract on cell viability of astrocytoma grade IV patient-derived glioma cells and U87MG cells after 24 h. Data were obtained from three independent experiments and are represented as mean ± SD. Not significant differences at *p* < 0.05 between patient-derived glioma cells versus U87MG cells

To determine the cytotoxic effect of *Rc* TR extract against grade IV human glioma cells (patient-derived and U87MG cells), the MTT assay was used. No significant differences were observed between patient-derived glioma cells and U87MG cells with regard to their viability (*p* < 0.05) (Fig. 1). *Rc* TR extract reduced the viability of both cell lines in a dose-dependent manner (0.1–3.0 mg/mL), reaching about 50% at a concentration of 1.0 mg/mL (Fig. 1).

### *Rc* TR extract induced double strand DNA breaks in the glioma cell lines and increased the numbers of phosphorylated H2A.X- and cleaved PARP1-positive cells

**Fig. 2:**
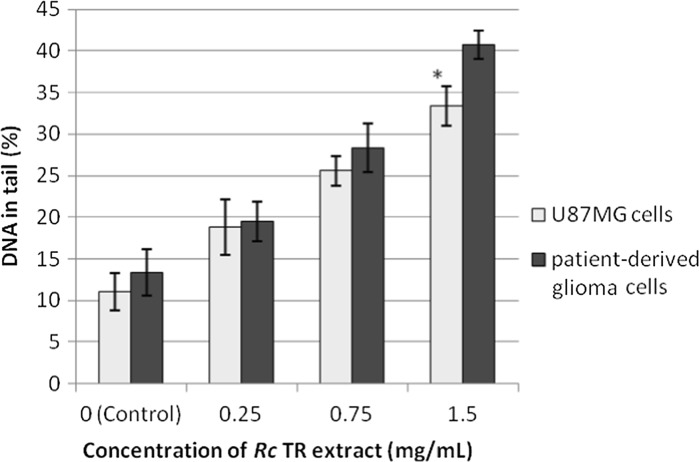
Double strand DNA damage in astrocytoma grade IV patient-derived glioma cells and U87MG cells after 24 h treatment with *Rc* TR extract measured by neutral comet assay. Each value represents mean ± SD from 3 independent experiments. *p < 0.05 patient-derived glioma cells versus U87MG cells

The study also used neutral comet assay to examine the ability of the *Rc* TR extract to induce DNA double strand breaks after 24-h treatment of glioma cell lines. It was found that the percentage of DNA damage increased with the concentration of *Rc* TR extract (Fig. 2), and the extract at the highest tested concentration (1.5 mg/mL) induced more DNA damage (significant differences *p* < 0.05) in the patient-derived glioma cells than in the U87MG cells. The percentages of DNA in the tail, were 41 and 34% for patient-derived glioma cells and U87MG cells, respectively (Figs. 2 and S1).

**Fig. 3:**
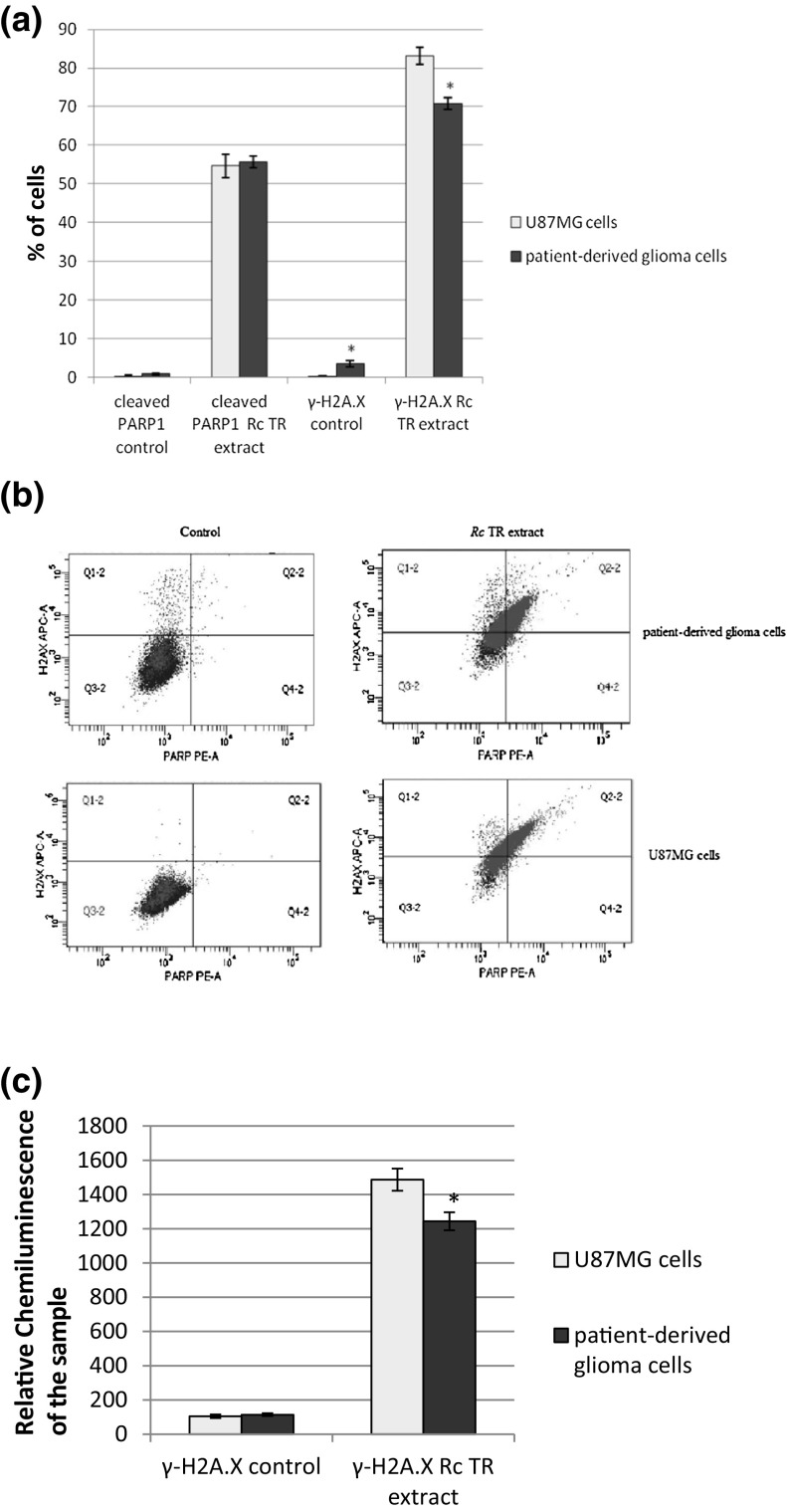
*Rc* TR extract increased the numbers of cleaved PARP1- and γ-H2A.X-positive cells. **a** The graph presents the percentage of cleaved PARP1- and γ-H2A.X-positive patient-derived glioma cells and U87MG cells  measured by flow cytometric analysis after 24 h treatment with *Rc* TR extract. **b** Representative flow cytograms. Control sample cells of both patient-derived glioma cells and U87MG cell line are located predominantly in bottom left quarter of the graph (cleaved PARP-negative, γ-H2A.X-negative). After treatment with *Rc* TR extract both samples appear at PAPR-positive and γ-H2A.X-positive areas of the graph indicating growth of dead cell population and ongoing DNA-repair processes. **c** The graph presents the level of γ-H2A.X after 24 h treatment of patient-derived glioma cells and U87MG cells with *Rc* TR extract, measured by Elisa test. Results represent mean ± SD from 3 independent experiments. *p < 0.05 patient-derived glioma cells versus U87MG cells

As the level of double-stranded breaks in the DNA was associated with the level of phosphorylated H2A.X, flow cytometry was used to measure the content of γ-H2A.X-positive cells. The percentage of cells expressing γ-H2A.X increased in both tested glioma cell lines after treatment with *Rc* TR extract in comparison to the control cells not treated with *Rc* TR extract, and was significantly higher (*p* < 0.05) for U87MG cells (about 83%) than for patient-derived glioma cells (about 70%) (Fig. 3a, b). The level of γ-H2A.X was also measured by Elisa test. Our findings showed that *Rc* TR extract increased the level of γ-H2A.X in both tested cancer cell lines and was higher in U87MG cells than in patient-derived glioma cells (Fig. 3c).

Flow cytometry was also used to estimate the percentage of cells expressing the apoptosis marker, i.e. cleaved Poly ADP-Ribose Polymerase 1 (PARP1), after 24-h incubation with *Rc* TR extract. It was found that the percentage of cleaved PARP1-positive cells was elevated in patient-derived glioma cells and U87MG cells compared to the control cells. The level of cleaved PARP1-positive cells remained similar in both glioma cell lines and was about 55% (Fig. 3a, b).

### *Rc* TR extract down-regulated the mRNA levels of UHRF1 and DNMT1

**Fig. 4:**
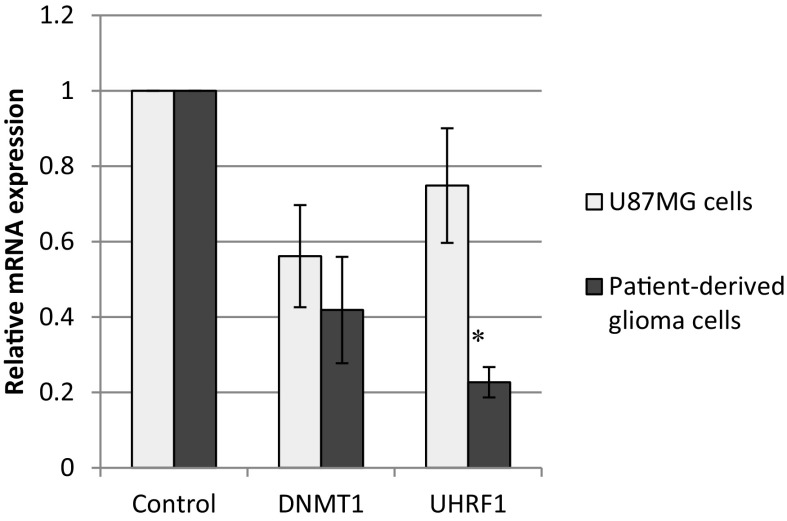
The expression of  *UHRF1* and *DNMT1* genes in patient-derived glioma cells and U87MG cells after 24 h treatment with *Rc* TR extract. Relative amount of mRNA was normalized to the 18S rRNA content. Data are presented as fold changes in patient-derived glioma cells or U87MG cells treated with *Rc* TR extract versus the cells non-treated with TR extract, in which expression levels of the genes were set as 1. Results are presented as mean ± SD of three independent experiments. *p < 0.05 patient-derived glioma cells versus U87MG cells

The real-time PCR was used to determine the mRNA expression of two genes, *UHRF1* and *DNMT1*, which are the DNA epigenetic markers. The mRNA levels of UHRF1 and DNMT1 were found to be reduced in both glioma cell lines treated with *Rc* TR extract (Fig. 4). The results showed significant differences (*p* < 0.05) in the level of *UHRF1* gene expression between both types of tested glioma cells (Fig. 4).

## Discussion

Our earlier studies showed that *Rhaponticum carthamoides* transformed root extract possessed anticancer potential; this acts by inducing apoptosis via the ROS-mediated mitochondrial pathway and caspase activation in grade II, III and IV human glioma cells obtained from patients (Skała et al. 2016, 2018). The present study was performed to better understand the anticancer mechanism of *Rhaponticum carthamoides* transformed root extract in grade IV human glioma cells, both those derived from patient and U87MG cells. Its aim was to determine whether the *Rc* TR extract is cytotoxic for the glioma cells, and whether it decreases the viability of these cells by inducing DNA damage, increasing the number of cleaved PARP1-positive cells and altering the level of γ-H2A.X-positive cells: a marker of double strand breaks in DNA. In addition, to determine whether *Rc* TR extract could induce the apoptosis of glioma cells, the levels of two DNA epigenetic markers, UHRF1 and DNMT1, were evaluated.

Many plant extracts or plant-derived compounds are known to play important roles as chemotherapeutic agents against various types of cancer (Gali-Muhtasib et al. 2015; Seca and Pinto 2018). One such group of compounds are the polyphenols with phenolic acids, which are known to demonstrate cytotoxic and anticancer activity (Anantharaju et al. 2016; Mojzer et al. 2016). These activities are associated with their structure: the presence of aromatic ring and hydroxylic groups. Additionally, the compounds with a higher number of hydroxylic groups exhibited better anticancer activity compared to the ones with no hydroxylic groups or compounds with –OCH3 moieties (Anantharaju et al. 2016). Additionally, different phenolic compounds may demonstrate a synergistic effect, both with each other and with well-known anticancer drugs used in clinical trials (Kuno et al. 2012; Mojzer et al. 2016). Polyphenols may induce apoptosis in cancer cells through the regulation of the genes involved in apoptosis, they may induce DNA damage, modulate ROS level or arrest the cell cycle (Anantharaju et al. 2016; Srivastava et al. 2016). Earlier studies have found *R. carthamoides* TR extract to be rich in phenolic acids, with tricaffeoylquinic acid derivatives (45.2 μg/mg) as the major fraction; the dominant phenolic acid was tentatively identified as tricaffeoylquinic acid derivative (33.7 µg/mg). The other main compounds were chlorogenic acid (28.9 µg/mg) and 3,5-*O*-dicaffeoylquinic acid (17.4 µg/mg) (Skała et al. 2015, 2016). Additionally, the *Rc* TR extract contained flavonoid glycosides such as quercetin, quercetagetin, luteolin, and patuletin hexosides (Skała et al. 2015). Caffeoylquinic acid derivatives possess anticancer activity against several types of cancer (Yoshimoto et al. 2002; Mishima et al. 2005; Kurata et al. 2007; Puangpraphant et al. 2011; Rocha et al. 2012; Weng and Yen 2012). Flavonoids are also known to possess anticancer properties (Batra and Sharma 2013; Mojzer et al. 2016). This is in accordance with our present findings, as well as those of Skała et al. (2016, 2018), and suggest that *R. carthamoides* transformed root extract, rich in caffeoylquinic acid derivatives and flavonoids, has the potential for cancer therapy. Previous studies indicate that *R. carthamoides* transformed root extract reduces the viability of glioma cells of various grades (II–IV grades) obtained from patients and induces apoptosis of these cells. The induction of apoptosis in cancer cells plays an important role in anticancer therapy (Hwang et al. 2015). It is worth noting that the transformed root extract of *R. carthamoides* did not demonstrate any cytotoxic activity against normal human astrocytes (Skała et al. 2016, 2018). Our present findings indicate that *Rc* TR extract reduces survival of grade IV glioma cells, both patient-derived cells and U87MG cells, in a dose-dependent manner.

It has previously been found that *R. carthamoides* TR extract is an efficient inducer of apoptosis in patient-derived grade IV glioma cells (Skała et al. 2016): The total count of early and late apoptotic cells after 24-h treatment with the highest concentration of this extract was about 30%. The previous studies (Skała et al. 2016, 2018) and current findings demonstrate that *R. carthamoides* transformed root extract may mediate apoptosis and the death of glioma cells through multiple pathways. The *Rc* TR extract may trigger apoptosis by the generation of ROS and the increase of Bax/Bcl-2 ratio and p53 mRNA levels, leading to the loss of mitochondrial membrane potential and subsequent caspase activation (Skała et al. 2016, 2018). A key novel finding of the present study was that treatment of glioma cells with *Rc* TR extract resulted in an increase in the number of the cleaved PARP1-positive glioma cells compared to the control cells, i.e. those not treated with *Rc* TR extract; this suggests that *Rc* TR extract containing phenolic acids and flavonoids induced apoptosis through the involvement of caspase cascade and PARP inactivation. It is known that the activation of executioner caspases can lead to cleavage of the cytoskeletal and nuclear proteins, including DNA repair enzymes, or lead to the activation of DNAses which cleave DNA in the nucleus (Kuno et al. 2012). It was found that polyphenolic compounds may mediate apoptosis by caspase activation and PARP1 cleavage; for example, *Corylus avellana* extract contains high amounts of polyphenols induces apoptosis in human malignant melanoma (SK-Mel-28) and human cervical cancer (HeLa) cell lines by this mechanism (Esposito et al. 2017). Caspase-dependent apoptosis with inactivation of PARP1 has also been observed after treatment of human lymphoblastoid TK6 cells with digallic acid obtained from *Pistacia lentiscus* fruits (Bhouri et al. 2012) or human colon cancer HCT-116 cells treated with green coffee bean extract (chlorogenic acid complex) (Gouthamchandra et al. 2017).

Various anticancer compounds can intercalate with DNA, leading to DNA damage and apoptotic cell death. One such group of DNA intercalators are the polyphenolic compounds with the flavonoids (Srivastava et al. 2016). In the present study, it was found that *Rc* TR extract has the ability to induce DNA double strand breaks in human glioma cells after 24-h treatment. This plant extract induced more DNA damage in glioma cells obtained surgically from a patient (41%) than in U87MG cells (34%). This increase of DNA damage in glioma cells after treatment with *Rc* TR extract may be due to DNA intercalation and PARP cleavage, which would be consistent with the results of many previous studies. For example, DNA damage in human glioma U373 cells treated with *Limoniastrum guyonianum* polyphenolic extract was associated with the PARP1 cleavage (Moniura et al. 2014). Similarly, *Moringa oleifera* extract, rich in polyphenols, induced DNA damage and inactivated PARP1 in human esophageal cancer cells (Tiloke et al. 2016). In addition, treatment with quercetin, one of the most common flavonoids belonging to phenolic compounds, also elevated PARP1 cleavage in Nalm6 leukemic cell lines (Srivastava et al. 2016).

As phosphorylation of H2A.X at Ser139 is also observed in response to DNA double-strand breaks (Kaur et al. 2006), the present study also measured by the flow cytometry the content of γ-H2A.X-positive cells in the population of patient-derived glioma cells and U87MG cells after treatment with *Rc* TR extract. The percentage of cells expressing phosphorylated H2A.X increased in comparison to the untreated, control cells in both tested glioma cell lines; however, the level was higher for U87MG cells (about 83%) than for patient-derived glioma cells (about 70%). Our results are consistent with those of previous studies: Resveratrol, a natural polyphenol, caused the formation of γ-H2A.X foci in HCT-116 colon carcinoma cells, which marked double-strand breaks in DNA (Demoulin et al. 2015). Also, chlorogenic acid, a common phenolic acid, induced DNA damage with increased levels of phosphorylated H2A.X in K562 leukemia cells (Burgos-Morón et al. 2012).

UHRF1, a ubiquitin-like protein with PHD and RING Finger domains 1 can participate in the repair of DNA damage (Sidhu and Capalash 2017; Tien et al. 2011). This protein appears at the DNA damage sites (Tien et al. 2011) and it directly recruits DNMT1 to hemi-methylated DNA (Sidhu and Capalash 2017). UHRF1 depletion inhibits connection of DNMTI with chromatin, resulting in hypomethylation of different genes (Tien et al. 2011). The down-regulation or silencing of UHRF1 in cancer cells resulted in the increase of DNA damage and the inhibition of cancer cell proliferation, as well as cell cycle arrest and caspase-8 dependent apoptosis (Tien et al. 2011), and up-regulation of p53 or p73 (Sidhu and Capalash 2017). Moreover, Tien et al. (2011) demonstrated an increased level of γ-H2A.X and PARP1 cleavage in UHRF1-depleted cells. Our findings indicate that the mRNA levels of UHRF1 and DNMT1 were reduced in glioma cell lines treated with *Rhaponticum carthamoides* TR extract. The polyphenol-rich *Aronia melanocarpa* juice and *Vaccinium myrtillus* extract (Antho 50) also showed down-regulation of UHRF1 in Jurkat cells and chronic lymphocytic leukemia cells, respectively (Sharif et al. 2012; Alhosin et al. 2015). Similarly, after treatment with *Limoniastrum guyonianum* polyphenolic extract, a decrease in UHRF1 and in the subsequent DNMT1 expression was observed in HeLa cells (Krifa et al. 2013). Down-regulation of DNMT1 was also described in melanoma cells and MCF-7 breast cancer cells after treatment with curcumin, a natural phenol (Smith et al. 2015).

## Conclusion

In conclusion, *Rhaponticum carthamoides* transformed root extract, which is rich in caffeoylquinic acid derivatives, may be a promising source of a novel anticancer agent. Our findings confirm those of previous studies, indicating that *R. carthamoides* transformed root extract may mediate apoptosis and the death of glioma cells through multiple pathways. This study reveals for the first time that the *Rc* TR extract causes cleavage and inactivation of PARP1, and/or inhibition of its synthesis, resulting in greater DNA damage and an elevated level of phosporylated H2A.X. Additionally, this extract decreases the level of DNA epigenetic markers such as UHRT1 and DNMT1. The findings of present and earlier studies suggest that * R. carthamoides* root extract may be of great importance in the anticancer drug development; however, further in vitro studies on other cancer cell lines and studies in animal models are necessary, either alone or in combination with known anticancer drugs.

## Electronic supplementary material

Below is the link to the electronic supplementary material.

Supplementary material 1 (DOCX 113 kb)
