# Supplementary material for: Rhaponticum carthamoides transformed root extract inhibits human glioma cells viability, induces double strand DNA damage, H2A.X phosphorylation, and PARP1 cleavage
Source: Cytotechnology. 2018 Aug 31;70(6):1585–94. doi: 10.1007/s10616-018-0251-3 (PMC6269353; doi:10.1007/s10616-018-0251-3)
Supplement: Supplementary file 1 — Supplementary material 1 (DOCX 113 kb) [file 10616_2018_251_MOESM1_ESM.docx]

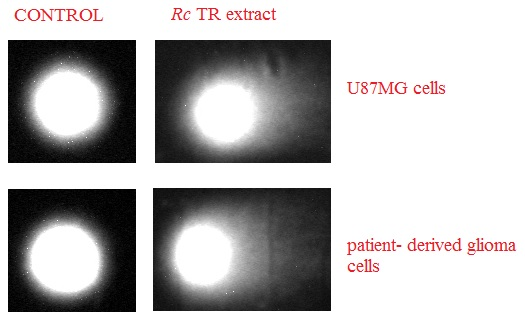


**Fig. S1.** Representative images of comet assay in U87MG cells and IV grade patient-derived glioma cells in control, untreated cells and after 24h treatment with *Rc* TR extract.
